# Inhibition of Pertussis Toxin by Human α-Defensins-1 and -5: Differential Mechanisms of Action

**DOI:** 10.3390/ijms241310557

**Published:** 2023-06-23

**Authors:** Carolin Kling, Anja Sommer, Yasser Almeida-Hernandez, Armando Rodríguez, Julio A. Perez-Erviti, Rajendra Bhadane, Ludger Ständker, Sebastian Wiese, Holger Barth, Mario Pupo-Meriño, Arto T. Pulliainen, Elsa Sánchez-García, Katharina Ernst

**Affiliations:** 1 Institute of Experimental and Clinical Pharmacology, Toxicology and Pharmacology of Natural Products, Ulm University Medical Center, 89081 Ulm, Germany; 2 Computational Bioengineering, Fakultät Bio- und Chemieingenieurwesen, Technische Universität Dortmund, 44227 Dortmund, Germany; 3 Core Facility Functional Peptidomics, Faculty of Medicine, Ulm University, 89081 Ulm, Germany; 4 Core Unit Mass Spectrometry and Proteomics, Faculty of Medicine, Ulm University, 89081 Ulm, Germany; 5 Institute of Biomedicine, University of Turku, FI-20520 Turku, Finland; 6 Departamento de Bioinformática, Centro de Matemática Computacional, Universidad de las Ciencias Informáticas (UCI), Havana 19370, Cuba

**Keywords:** pertussis toxin, defensins, bacterial AB-type toxin, human peptide, antimicrobial peptide, toxin inhibitor, mechanism of inhibition

## Abstract

Whooping cough is a severe childhood disease, caused by the bacterium *Bordetella pertussis*, which releases pertussis toxin (PT) as a major virulence factor. Previously, we identified the human antimicrobial peptides α-defensin-1 and -5 as inhibitors of PT and demonstrated their capacity to inhibit the activity of the PT enzyme subunit PTS1. Here, the underlying mechanism of toxin inhibition was investigated in more detail, which is essential for developing the therapeutic potential of these peptides. Flow cytometry and immunocytochemistry revealed that α-defensin-5 strongly reduced PT binding to, and uptake into cells, whereas α-defensin-1 caused only a mild reduction. Conversely, α-defensin-1, but not α-defensin-5 was taken up into different cell lines and interacted with PTS1 inside cells, based on proximity ligation assay. In-silico modeling revealed specific interaction interfaces for α-defensin-1 with PTS1 and vice versa, unlike α-defensin-5. Dot blot experiments showed that α-defensin-1 binds to PTS1 and even stronger to its substrate protein Gαi in vitro. NADase activity of PTS1 in vitro was not inhibited by α-defensin-1 in the absence of Gαi. Taken together, these results suggest that α-defensin-1 inhibits PT mainly by inhibiting enzyme activity of PTS1, whereas α-defensin-5 mainly inhibits cellular uptake of PT. These findings will pave the way for optimization of α-defensins as novel therapeutics against whooping cough.

## 1. Introduction

*Bordetella (B.) pertussis* toxin (PT) is an AB_5_ protein toxin consisting of an enzyme subunit, the A-protomer PTS1, and a pentameric B-subunit, which together form a holotoxin with a pyramid-like structure that is secreted by the bacteria [1,2,3]. The B-pentamer allows binding to the cell surface via sialoglycoproteins present on most cell types [4,5]. Following internalization by endocytosis, PT undergoes retrograde intracellular transport through the Golgi to the endoplasmic reticulum (ER) [6,7]. In the ER, ATP binding to the toxin causes destabilization of the interaction between PTS1 and the B-pentamer, leading to the release of PTS1 from the holotoxin [8,9,10,11]. The released PTS1 is thermally unstable and therefore detected by the ER-associated degradation (ERAD) pathway, which transports it from the ER to the cytosol [11,12]. PTS1 lacks lysine residues, which protects it from ubiquitination and proteasomal degradation [13]. Furthermore, host cell chaperones and protein folding helper enzymes are required for the uptake of PTS1 into the host cell cytosol [14,15,16,17,18].

Cytosolic PTS1 covalently transfers an ADP-ribose moiety from its co-substrate NAD^+^ onto the α-subunit of inhibitory G-proteins (Gαi), which are associated with G-protein-coupled receptors (GPCRs) [19,20]. ADP-ribosylation inactivates Gαi, resulting in disturbed cAMP signaling, as Gαi is no longer able to inhibit the adenylate cyclase (AC). The consequences of disturbed GPCR and cAMP signaling are varied and depend on the cell type (for review see also [21]). For example, PT was first referred to as ‘islet-activating protein’ because it stimulates insulin secretion [20,22]. Moreover, in the early stages of infection, PT inhibits the recruitment of neutrophils, monocytes, and lymphocytes to the lung, leading to a decrease in pro-inflammatory chemokines and cytokines, as well as an increased bacterial burden in a mouse model [23,24,25]. PT also reduces the surface expression of adhesion molecules and integrins (leukocyte function antigen-1, LFA-1; L-selectin) resulting in disturbed trafficking of lymphocytes and therefore trapping of lymphocytes in the vasculature [26,27].

PT is a significant virulence factor responsible for the symptoms of whooping cough, a disease characterized by a prolonged (about ten weeks) and uncontrollable cough [28,29]. Patients often experience secondary complications, including vomiting and pneumothorax. Severe cases can lead to pneumonia, encephalopathy, seizures, or apnea, which can be life-threatening, particularly in newborns and young infants [30]. According to the World Health Organization (WHO), in 2014, over 24.1 million cases of pertussis in children under five years old resulted in 160,700 deaths worldwide [31,32]. Pertussis is re-emerging despite available vaccination and high vaccination coverage in Western countries. Case numbers are at an all-time high since vaccine introduction in the 1950s [31,33]. Although antibiotic therapy eliminates *B. pertussis* bacteria, it does not relieve pertussis symptoms, except when administered within two weeks of infection, which rarely occurs due to late diagnosis [28]. Studies have demonstrated that PT causes prolonged and severe inflammation of the airways in a mouse model [34]. In fact, strains of *B. pertussis* not expressing PT did not cause severe symptoms such as leukocytosis or death, indicating the crucial role of PT especially in severe cases [21]. As a result, PT represents an attractive target for the development of novel therapeutic strategies.

Recently, we identified the human antimicrobial peptides α-defensin-1 and α-defensin-5, also known as human neutrophil peptide 1 (HNP1) and human defensin-5 (HD5), respectively, as inhibitors of PT [35]. Defensins are small, cationic, and cysteine-rich peptides with a crucial role in innate immunity by inactivating bacterial pathogens [36]. α-Defensin-1 and α-defensin-5 but not the structurally related ß-defensin-1 protected cells from PT-intoxication and from PT-mediated effects on intracellular cAMP signaling. We showed that α-defensin-1 and -5 inhibited the enzyme activity of PTS1 in vitro [35]. In the present study, the underlying inhibitory mechanism of α-defensin-1 and -5 on the mode of action and uptake mechanism of PT was characterized in detail to elucidate innate immune defense mechanisms against PT as well as to provide a starting point for novel therapeutic strategies against PT-mediated symptoms of whooping cough.

## 2. Results

### 2.1. Human α-Defensin-1 and -5 Inhibit ADP-Ribosylation of Gαi in PT-Treated Cells and Enzyme Activity of PTS1 In Vitro

**Figure 1:**
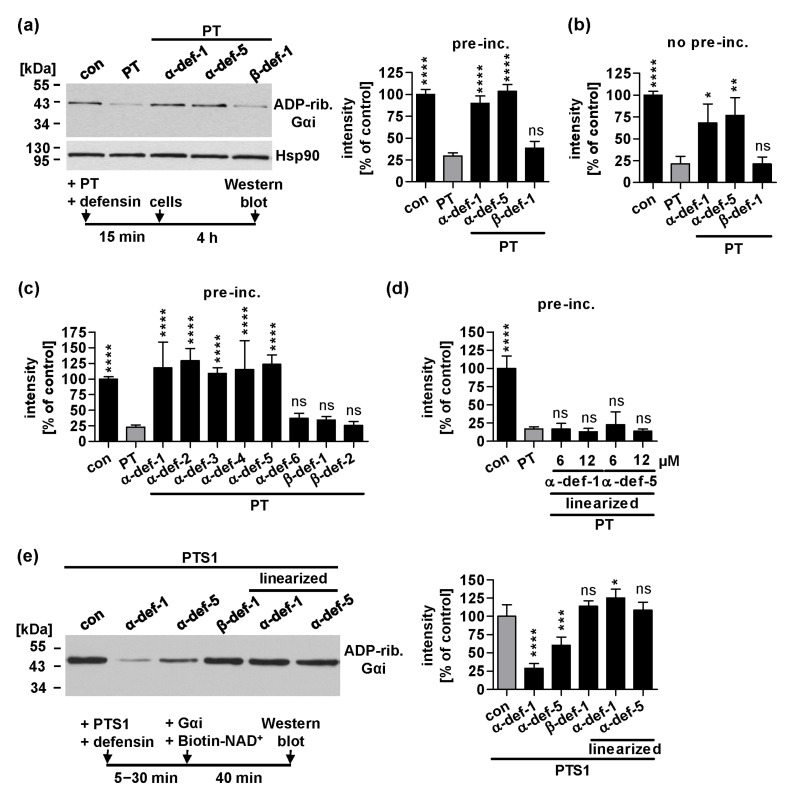
Effect of defensins on ADP-ribosylation status of Gαi in PT-treated cells and enzyme activity of PTS1 in vitro (**a**) PT (50 ng/mL) was pre-incubated with defensin (12 μM) or with the respective amount of solvent (H_2_O) for 15 min at room temperature and then added to A549 cells for 4 h at 37 °C. Cells were left untreated for a further control (con). Subsequently, cells were lysed, and Gαi, which has not been ADP-ribosylated in cells during intoxication was ADP-ribosylated and therefore biotin-labeled by incubation with PTS1 and biotin-labeled NAD^+^. Biotin-labeled Gαi was detected via Western blot. Hsp90 was detected as a control of equal protein loading. The bar graph shows quantified Western blot signals from four independent experiments. Values are given as percent of untreated control, normalized to Hsp90, mean ± SEM (n = 4 from four independent experiments) (**b**) PT (50 ng/mL) and defensin (12 μM) or the respective amount of solvent (H_2_O) were added directly to A549 cells and incubated for 4 h at 37 °C. Subsequently, the experiment was performed as described in (**a**). (**c**,**d**) PT (10 ng/mL) was pre-incubated with defensins (6 μM (**c**) or as indicated (**d**)) for 15 min at room temperature and added to CHO-K1 cells and incubated for 4 h at 37 °C. Subsequently, the experiment was performed as described in (**a**). (**c**) Values in bar graphs are given as percent of untreated control, normalized to Hsp90, mean ± SEM (n ≥ 4 from at least four independent experiments). (**d**) Values in bar graphs are given as percent of untreated control, normalized to Hsp90, mean ± SD (n = 3 from three independent experiments). (**e**) PTS1 (50 nM) was pre-incubated with 6 μM defensin or the respective amount of solvent (H_2_O) for 5–30 min at 37 °C. Gαi and biotin-labeled NAD^+^ were added for 40 min at room temperature. ADP-ribosylated and therefore biotin-labeled Gαi was detected via Western blot. The bar graph shows quantified Western blot signals from at least three independent experiments. Values are given as percent of control, mean ± SEM (n ≥ 6 from at least four independent experiments. (**a**–**e**) Significance was tested using one-way ANOVA followed by Dunnett’s multiple comparison test and refers to controls treated with PT only ((**a**–**d**), grey bars) or untreated controls ((**e**), grey bars) (* *p* ≤ 0.1, ** *p* ≤ 0.01, *** *p* ≤ 0.001 **** *p* ≤ 0.0001, ns not significant).

As we previously demonstrated the inhibitory effects of α-defensin-1 and -5 on CHO-K1 and HEK293 cells [35], we now confirmed this finding in the lung adenocarcinoma cell line A549, which is a more relevant cell line regarding the pathophysiology of infection and PT secretion. A549 cells were treated with PT for 4 h, either after pre-incubation of PT with the defensin (Figure 1a) or direct administration (Figure 1b). Afterwards, the ADP-ribosylation status of Gαi in the cell lysate was determined by sequential ADP-ribosylation: Gαi, which has not been ADP-ribosylated in cells during intoxication was biotin-labeled by incubation with PTS1 and biotin-NAD^+^. Thus, samples from PT-treated cells show a weaker signal in the Western blot than samples from untreated cells (con). In the presence of α-defensin-1 or -5, the PT-mediated ADP-ribosylation of Gαi in A549 cells was inhibited, whereas β-defensin-1 had no effect (Figure 1a,b).

In addition, other defensins of the same family were investigated in this assay, and α-defensin-2, -3 and -4 exhibited the same inhibitory effect as α-defensin-1 and -5 (Figure 1c). In contrast, α-defensin-6, along with β-defensin-1 and -2, had no effect on the PT-induced ADP-ribosylation of Gαi. Moreover, the common characteristic disulfide bonds of human α-defensins were disrupted by replacing cysteine residues with serine residues in their amino acid sequence. These thereby linearized peptides α-defensin-1 and -5, lacking disulfide bonds, were unable to reduce the levels of ADP-ribosylated Gαi in PT-treated cells, even in higher concentrations (Figure 1d). This demonstrates the importance of the tertiary structure of α-defensin-1 and -5 to inhibit PT.

The enzyme activity of PTS1 was assessed separately by pre-incubating PTS1 with the respective defensins and then adding Gαi and biotin-NAD^+^ to the in vitro reaction (Figure 1e). ADP-ribosylated and thus biotin-labeled Gαi was detected by Western blot, and a reduction in the signal indicates inhibition of PTS1 activity. The linearized α-defensin-1 and -5 did not exhibit inhibitory activity against PTS1, whereas their native forms demonstrated a clear reduction in ADP-ribosylated Gαi. Notably, α-defensin-1 exhibits a stronger inhibition of PTS1 enzyme activity than α-defensin-5.

### 2.2. α-Defensin-5 Reduces the Amount of Cell-Bound PT Stronger Than α-Defensin-1

**Figure 2:**
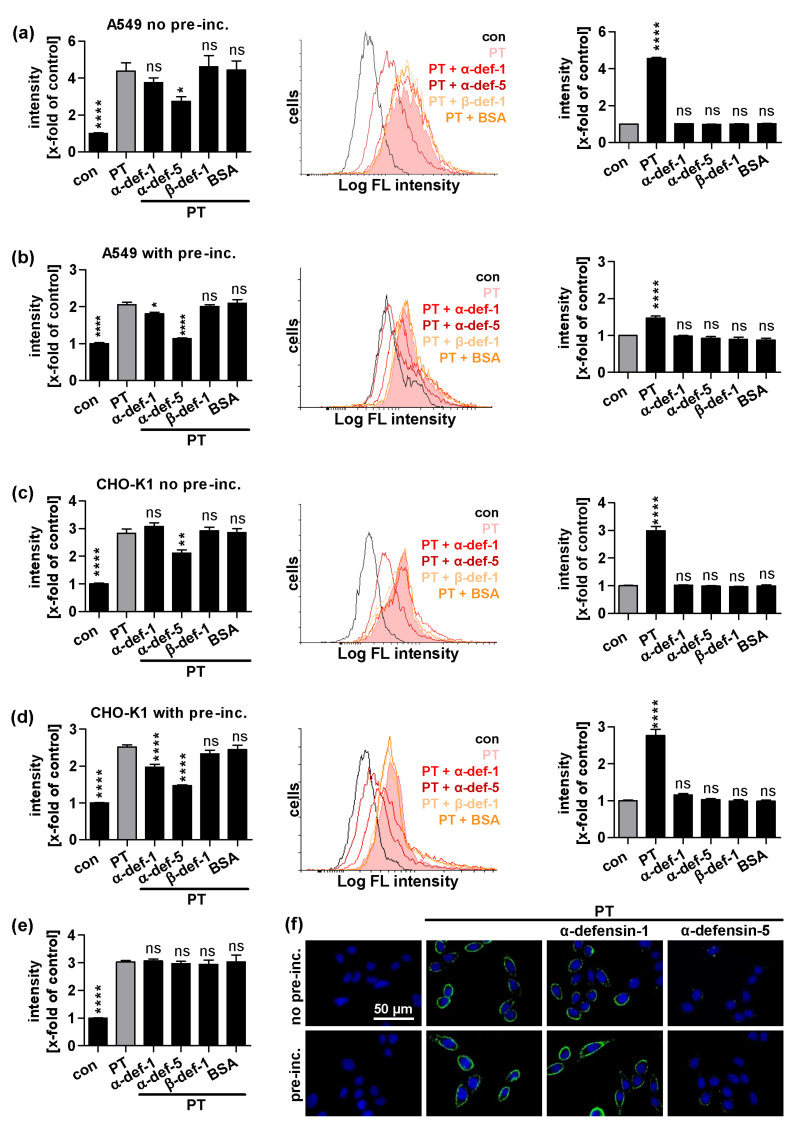
Effect of defensins on binding of PT to cells. PT that was 488-labeled (1 μg/mL (**a**,**b**) or 0.5 μg/mL (**c**,**d**)) was pre-incubated with defensin (12 μM) for 15 min at room temperature (**b**,**d**) or added directly (**a**,**c**) to A549 or CHO-K1 cells in suspension and incubated for 15 min on ice to enable binding of PT to the cell surface. For control, cells were left untreated (con). Cells were washed by centrifugation and binding of PT was detected by flow cytometry. Values are given as percent of control, mean ± SEM (n = 9 from three independent experiments (left) or n = at least 3 from at least one experiment (right)). Histograms show fluorescence intensity of cells in one representative experiment, respectively. (**e**) PT that was 488-labeled (0.5 μg/mL) was pre-incubated with CHO-K1 cells for 15 min on ice. Cells were washed and incubated with defensin (12 μM) for 15 min on ice. For control, cells were left untreated (con). Subsequently, the experiment was performed as described above. Values are given as percent of control, mean ± SEM (n = 8 from three independent experiments) (**a**–**e**) Significance was tested using one-way ANOVA followed by Dunnett’s multiple comparison test and refers to controls treated with PT only (left, grey bars) or untreated controls (right, grey bars) (* *p* ≤ 0.1, ** *p* ≤ 0.01, **** *p* ≤ 0.0001, ns not significant). (**f**) PT (1 μg/mL) was pre-incubated with defensin (6 μM) for 15 min at room temperature (lower row) or added directly (upper row) to CHO-K1 cells and incubated for 40 min on ice to enable binding of PT to the cell surface. Cells were washed, fixed, permeabilized, blocked and probed with an antibody against PTS1 (green). Nuclei were stained with Hoechst (blue). Representative images of three individual experiments are shown.

Prompted by these findings, we further investigated the mechanism of inhibition of α-defensin-1 and -5. When cells are incubated with PT on ice, the toxin can only bind to the cell surface but is not taken up into the cells. We analyzed bound PT on A549 and CHO-K1 cells by flow cytometry (Figure 2a–d). In both A549 and CHO-K1 cells, α-defensin-1 only inhibited binding of PT to the cell surface when it was pre-incubated with PT before adding it to the cells (Figure 2b,d). However, when this pre-incubation step was omitted, α-defensin-1 showed no inhibitory effect (Figure 2a,c). α-Defensin-5 showed a clear and strong reduction in the amount of PT bound to the cell surface, regardless of whether pre-incubation was performed or not. β-Defensin-1 and the unrelated protein BSA served as negative controls and had no effect on PT binding to cells. α-Defensin-1 and -5 alone did not reduce the auto-fluorescence of the analyzed cells (Figure 2a–d on the right). Furthermore, neither α-defensin-1 nor -5 showed a reduction in the amount of PT bound to the cell surface when they were added to the cells after PT had already bound (Figure 2e).

As an alternative approach, the amount of PT bound to the cell surface after incubation on ice in the presence of α-defensin-1 and -5 was also investigated by fluorescence microscopy, utilizing a specific antibody against PT (Figure 2f). Here, the amount of PT on the cell surface was only reduced by α-defensin-5 but not α-defensin-1, regardless of whether pre-incubation was performed or not.

### 2.3. Treatment with α-Defensin-5 Results in a Reduction in the Amount of PT in Cells

**Figure 3:**
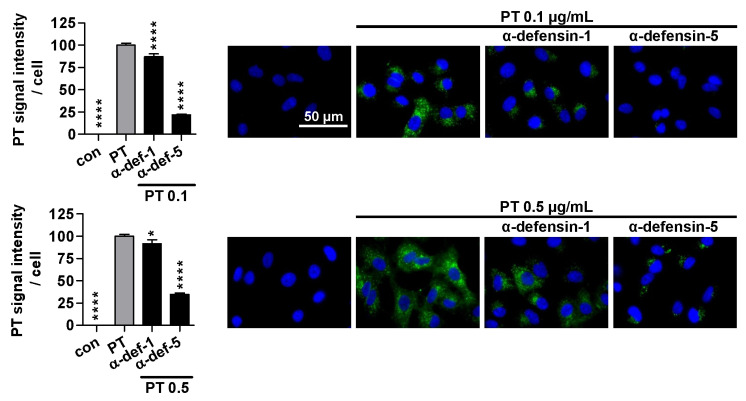
Effect of defensins on PT signals in cells. A549 cells were treated with PT (0.1 or 0.5 μg/mL) and defensin (6 μM) for 4 h at 37 °C. For control, cells were left untreated (con). Cells were washed, fixed, permeabilized, blocked and probed with an antibody against PT (green). Nuclei were stained with Hoechst (blue). Representative images of three independent experiments are shown. The overall intensity of the PT signal was determined with ImageJ v.1.52a. Values are baseline-corrected, normalized to cell numbers and given as mean ± SEM (n = 45 images per condition from three independent experiments). Significance was tested using one-way ANOVA followed by Dunnett’s multiple comparison test and refers to samples treated only with PT (grey bars) (* *p* ≤ 0.1, **** *p* ≤ 0.0001, ns not significant).

After observing the inhibitory effect of α-defensin-5 on PT binding to the cell surface, we proceeded to investigate the overall amount of PT in cells. Therefore, A549 cells were treated with PT at 37 °C for 4 h, and then the signal intensity of PT within the cells was analyzed (Figure 3). In the presence of α-defensin-1, the signal intensity of PT was only slightly reduced, whereas in the presence of α-defensin-5, a substantial decrease in signal intensity was observed.

### 2.4. α-Defensin-1 Is Internalized into Cells

**Figure 4:**
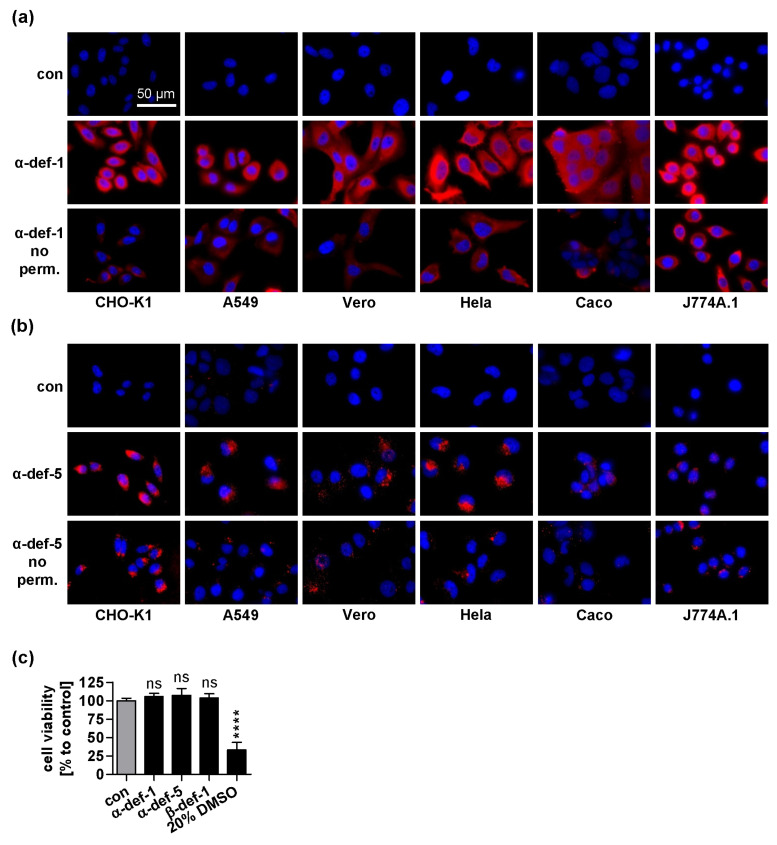
Uptake of defensins into cells. (**a**–**b**) Different cell lines were incubated with respective defensin (6 μM) for 4 h at 37 °C. Cells were washed, fixed and either permeabilized (upper rows) or this step was omitted (lower rows) to prevent intracellular staining with antibodies. Then, cells were blocked and probed with an antibody against α-defensin-1 (**a**) or α-defensin-5 (**b**) (both red). Nuclei were stained with Hoechst (blue). Representative images of three independent experiments are shown. (**c**) CHO-K1 cells were treated with defensin (6 μM) or 20 % DMSO as a positive control for decreasing cell viability for 24 h at 37 °C. Cell viability was measured by the MTS assay. Absorbance values are given as percent of untreated control, mean ± SEM (n = 9 from three independent experiments). Significance was tested using one-way ANOVA followed by Dunnett’s multiple comparison test and refers to non-treated samples (grey bars) (**** *p* ≤ 0.0001, ns not significant).

Our results so far indicate that α-defensin-5 mainly inhibits PT intoxication by preventing PT from entering cells. For α-defensin-1, however, the results suggest an intracellular mechanism of PT inhibition, such as inhibiting enzyme activity of PTS1 in cells. Hence, it is plausible to consider that α-defensin-1 has to reach its target in the cytosol of the cells to interact with and inhibit PTS1. Therefore, we examined whether α-defensin-1 was taken up into cells. Different cell lines were incubated with α-defensin-1 or -5 at 37 °C for 4 h (Figure 4a,b). Then, cells were fixed and the defensins were stained with specific antibodies. Permeabilization of cells allows the antibodies to reach intracellular targets. Performing the experiment with or without permeabilization allows distinguishing the intracellular and extracellular signals of defensins (Figure 4a,b, lower row). When the cells were incubated with α-defensin-1, we observed a strong signal with permeabilization and weaker signal without permeabilization indicating a primarily intracellular signal localization (Figure 4a). Incubation with α-defensin-5 resulted in a much weaker signal, which did not differ as much with or without permeabilization (Figure 4b). Despite the substantial uptake of α-defensin-1, it had no influence on cell viability even after incubation for 24 h (Figure 4c).

### 2.5. α-Defensin-1 but Not α-Defensin-5 Is Detected in Close Proximity to PTS1 in Cells

**Figure 5:**
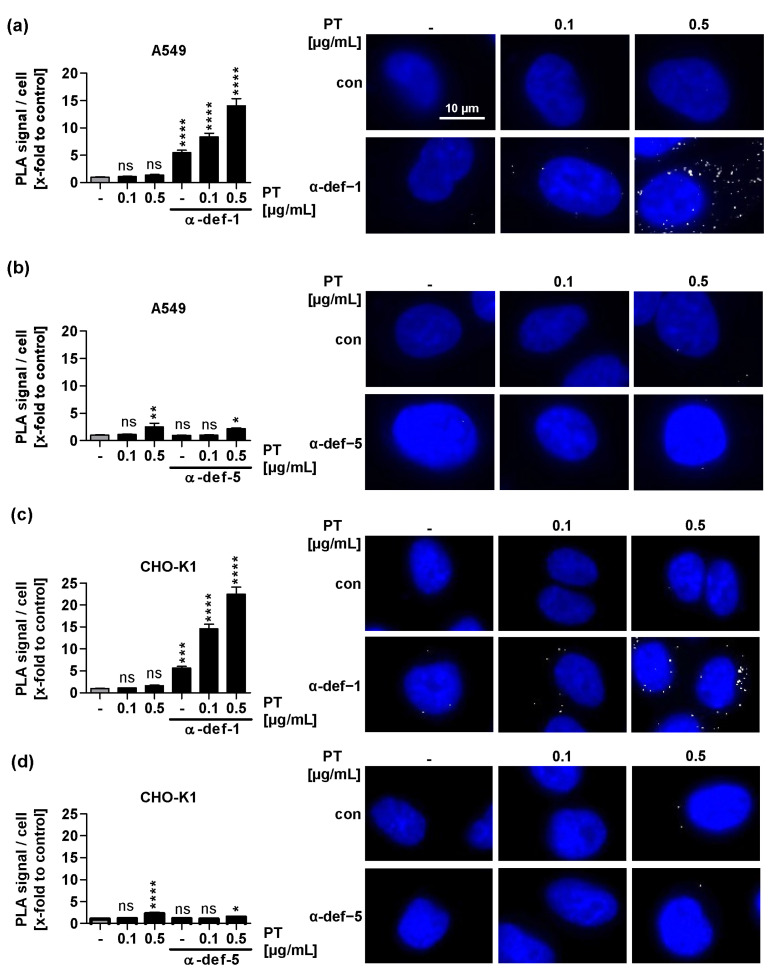
Interaction of defensins with PTS1 in cells. A549 (**a**,**b**) or CHO-K1 (**c**,**d**) cells were treated with PT and defensin (6 μM) for 4 h at 37 °C. After washing, cells were fixed, and the fluorescence-based PLA assay was performed according to the manufacturer’s manual. Nuclei were stained with Hoechst (blue). PLA signals (white) represent protein interaction events of PTS1 and α-defensin-1 (**a**,**c**) or α-defensin-5 (**b**,**d**) and were counted from fluorescence pictures with ImageJ v.1.52a. (**a**–**d**) Graphs show quantified PLA signals per cell number. Values are given as mean ± SEM (n = 45 images per condition from three independent experiments). Significance was tested using one-way ANOVA followed by Dunnett’s multiple comparison test and refers to untreated controls (grey bars) (* *p* ≤ 0.1, ** *p* ≤ 0.01, *** *p* ≤ 0.001, **** *p* ≤ 0.0001, ns not significant).

After demonstrating the uptake of α-defensin-1 into cells, we investigated whether it also interacts with PTS1 in cells. Therefore, the proximity ligation assay (PLA), which generates a fluorescent signal when two specific antibodies are in close proximity within fixed cells due to an internal amplification reaction was employed. A549 and CHO-K1 cells were treated with defensin and PT for 4 h at 37 °C (Figure 5). Then, cells were fixed, and the PLA was performed using antibodies against PTS1 and α-defensin-1 or -5, respectively. Even though the background PLA signals for α-defensin-1 in control cells (not treated with PT) were significantly higher than without α-defensin-1, PLA signals in cells treated with PT and α-defensin-1 were still considerably higher (Figure 5a,c). On the other hand, PLA signals in cells treated with PT and α-defensin-5 did not surpass the background signals obtained in cells only treated with PT (Figure 5b,d). This shows that α-defensin-1 but not α-defensin-5 was detected very close to PTS1 in cells.

### 2.6. Computational Modeling of the PTS1/α-Defensin-1 and PTS1/α-Defensin-5 Binding Interface

Next, to provide mechanistic insights at the molecular level, we studied the interaction of PTS1 with α-defensin-1 and α-defensin-5. We employed different approaches to identify the binding interface of the complexes in-silico.

**Figure 6:**
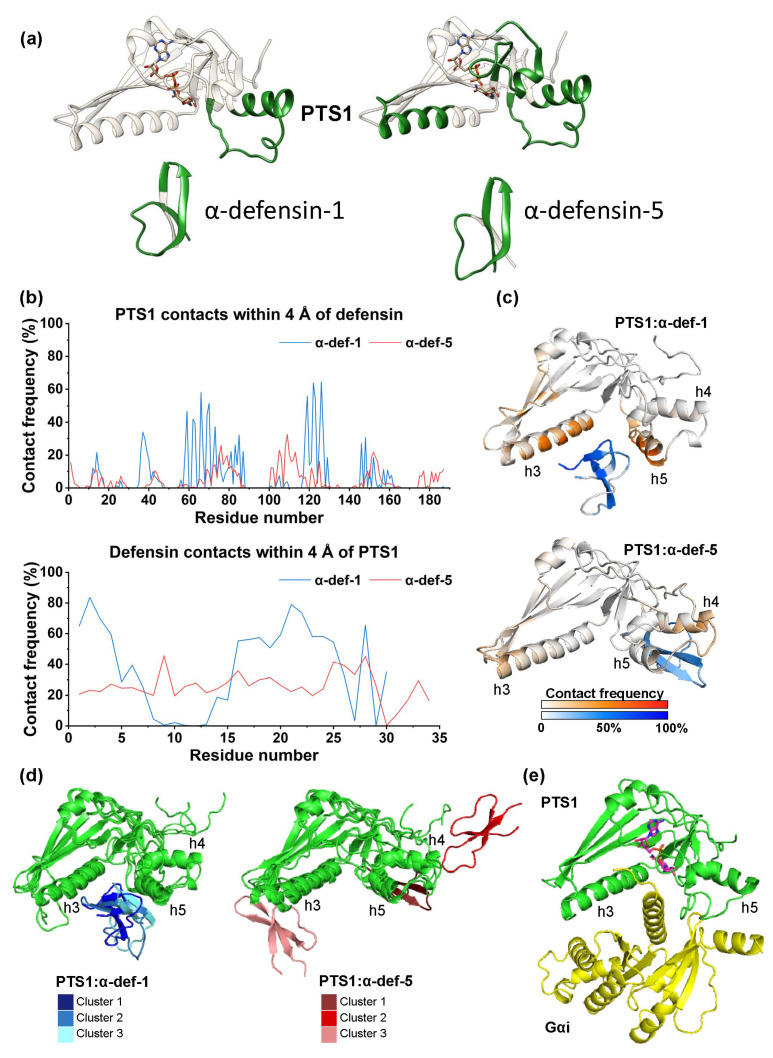
Computational modeling of the interaction of PTS1 with α-defensin-1 and -5 (**a**) PPI-Detect analysis of PTS1 and α-defensin-1 and -5. The stick representation shows BaAD, an NAD^+^ analog [39], on PTS1 (PDB ID 7SNE). The regions in green correspond to the top-10 subsequences with the highest interaction scores (within the applicability domain of the method). (**b**) Contact frequency (expressed as the percent of simulation time per residue with contacts < 4 Å) between PTS1 and α-defensin-1 (blue) or α-defensin-5 (red) during the last 800 ns of the simulations (averaged over the last 800 ns of each replica). (**c**) 3D representation of the most populated cluster in the combined last 800 ns of the three replicas of the simulations of PTS1/α-defensin-1 (**top**) and PTS1/α-defensin-5 (**bottom**). The color scale represents the mapped contact frequency per residue of PTS1 (white to red scale) and α-defensin-1 or α-defensin-5 (white to blue scale). (**d**) 3D representations of the three most populated clusters in the combined last 800 ns of the three replicas of PTS1/α-defensin-1 (left, PTS1 in green, α-defensin-1 in shades of blue for the different clusters) and PTS1/α-defensin-5 (right, PTS1 in green, α-defensin-5 in shades of red for the different clusters). (**e**) PTS1/Gαi complex modelled by Sakari et al. [39] using AlphaFold. PTS1 is shown in green, Gαi in yellow, and NAD^+^ in purple sticks.

First, we used PPI-Detect [37], a support vector machine model for the prediction of protein–protein interactions, to map the interaction probability of different regions in the sequence of each partner. The interaction scores were relatively higher for α-defensin-1 (0.636–0.604) than for α-defensin-5 (0.577–0.548) for the top-10 pairs of sequences inside the applicability domain. This result is in line with the experimental evidence that α-defensin-1 inhibition of PTS1 is stronger than for α-defensin-5 (Figure 1e). In both cases, the best-scored interactions were located mostly in the region of residues 101–130 of PTS1. In the case of α-defensin-5, the interactions with PTS1 were wider spread with respect to α-defensin-1 (Figure 6a). For both defensins, most of the peptide’s sub-sequences are involved in interactions with PTS1, except for the C-terminal region of α-defensin-1 and the N-terminal region of α-defensin-5.

Next, PTS1/α-defensin-1 and PTS1/α-defensin-5 complexes were modeled with AlphaFold Multimer (v2.2) [38] using the sequences of recombinant PTS1, α-defensin-1 and α-defensin-5 as inputs. This provided us with a structure of PTS1 based on the actual recombinant sequence used in the experiments. The models predicted the binding of both α-defensins in the region between α-helices h3 (residues 57–77) and h5 (residues 118–127) of PTS1 (Supplementary Figure S1). Furthermore, residues 118–127 of PTS1 are predicted to form a α-helix (h5), similar to the structure of the full toxin [1] and the auto-inhibited PTS1. This finding suggests that these residues might fold upon binding to a partner, unlike when PTS1 is not bound, when this region is unfolded [39].

Next, to account for the conformational flexibility of the protein and peptides and sample the conformational space of the systems, we carried out extensive molecular dynamics (MD) simulations. As a starting point for the MDs, we used the AlphaFold models with the α-defensins placed far away (40 Å) and in different orientations with respect to PTS1 (Supplementary Figure S2a). The simulation systems (PTS1/α-defensin) were allowed to move freely for 1.5 μs. Consequently, no binding sites or binding motifs were predefined. The simulation was repeated three times (replicas) for each PTS1/α-defensin system. During the first hundreds of nanoseconds of the simulations, the α-defensins diffused randomly in the solvent until binding to PTS1 was achieved. At 700 ns, both α-defensin-1 and α-defensin-5 were bound to PTS1 in all replicas (Supplementary Figure S2b, Supplementary Movies S1 and S2). Therefore, all subsequent analyses are based on the last 800 ns of each replica.

The analysis of the residues of the α-defensins and PTS1 featuring intermolecular contacts less than 4 Å away (Figure 6b,c) showed that both α-defensin-1 and α-defensin-5 display different binding modes to PTS1. In the PTS1/α-defensin-1 simulations, the interactions of α-defensin-1 with PTS1 mostly involved the PTS1 residues in α-helices h3 (residues 57–77) and h5 (residues 118–127). PTS1 residues with over 50% contact frequency with α-defensin-1 include H66 (58.0%), D70 (51.5%), L119 (55.9%), A122 (63.9%), L123 (53.4%) and Y126 (64.6%). As predicted using PPI-Detect, the residues of α-defensin-1 with more than 50% contact frequency with PTS1 are mainly located at the following regions of α-defensin-1: N-terminus (residues 1–4), β-sheet 2 (residues 16–21), the loop between β-sheet 2 and 3 (residues 22–24), and residues 25 and 28 of β-sheet 3. Conversely, α-defensin-5 interacts with several patches along the surface of PTS1: helix h4 and the C-terminal loop (100–114), the N-terminal region of segment h3 (residues 72–81), and the turn between β-sheets 8 and 9 (residues 151–154). Unlike α-defensin-1, α-defensin-5 does not display conserved interactions with specific residues of PTS1, and PTS1 does not target specific regions of α-defensin-5. 

To further clarify the binding mode between PTS1 and both defensins, we carried out root mean square deviation (RMSD) cluster analysis with 5 Å cutoff after combining the last 800 ns of the three replicas of each system. We observed no relevant changes in the secondary structure motifs of PTS1 upon binding of the defensins with respect to the starting structure of PTS1 generated by AlphaFold (Supplementary Figure S3). However, we found a different binding behavior for α-defensin-1 and α-defensin-5.

The three most populated clusters of the PTS1/α-defensin-1 simulation (Figure 6d, left) contain 50.4% of the analyzed trajectory frames. Representative structures of each cluster of α-defensin-1 bind to a cleft between helices h3 and h5, opposite to the NAD^+^ binding site. In the case of PTS1/α-defensin-5, the three more populated clusters contain 41.4% of the simulation frames (Figure 6d, right). However, the representative structures of each cluster evidence interactions with different regions of PTS1, unlike the case of α-defensin-1 (Figure 6d).

The MD simulations of PTS1 with α-defensin-1/α-defensin-5 in explicit water delivered different binding modes for each defensin. α-Defensin-1 shows a clear tendency to bind PTS1 at the cleft between α-helices h3 and h5, as predicted by the AlphaFold model. Contrarily, α-defensin-5 does not have a preferred binding site at PTS1. Accordingly, the unbiased simulations allow us to discard the AlphaFold model of α-defensin-5 bound to the h3/h5 cleft of PTS1 as the preferred binding region.

The observed binding mode of α-defensin-1 to PTS1 does not occlude the NAD^+^ binding site. Thus, the NAD^+^ hydrolase activity of PTS1 is not expected to be affected by α-defensin-1 binding. Although no crystallographic structure of PTS1 bound to its substrate Gαi is available, the PTS1/Gαi complex was modelled by Sakari et al. [39] (Figure 6e). In this model, the N-terminus of Gαi, which contains the cysteine residue that gets ADP-ribosylated by PTS1, inserts into the PTS1 active site via the cleft between helices h3 and h5 of PTS1. This occurs in a similar manner to the preferred binding mode of α-defensin-1 observed in our simulations. This implies that α-defensin-1 could sterically block the PTS1-catalyzed Gαi ADP-ribosylation.

### 2.7. α-Defensin-1 Directly Binds to PTS1 and Gαi In Vitro

**Figure 7:**
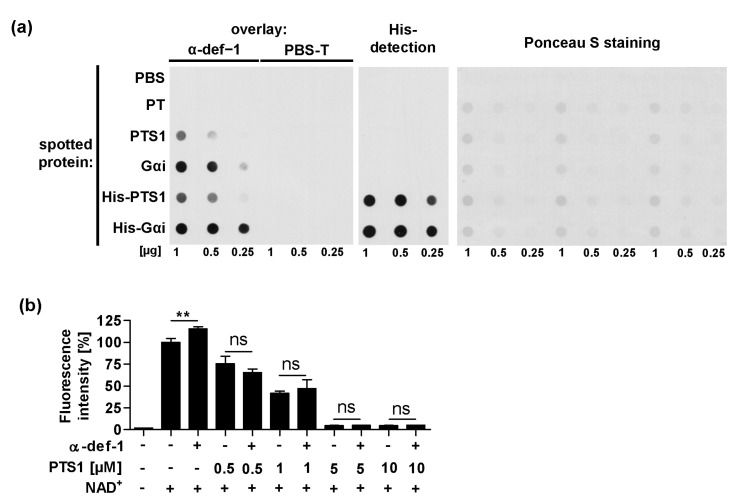
Interaction of α-defensin-1 with PTS1 and Gαi in vitro. (**a**) Dot blot assay. Decreasing amounts of proteins (1 μg, 0.5 μg, 0.25 μg) were spotted onto a membrane using the dot blot system. After blocking, the membrane was cut and probed with α-defensin-1 (0.4 μg/mL) or PBS-T for control. After extensive washing, bound α-defensin-1 was detected with an antibody against α-defensin-1 using the ECL system ((**left**) panel). The presence of the N-terminal 6x-His-tag was shown by an antibody against 6x-His-tag using the ECL system ((**middle**) panel). Comparable amounts of spotted protein were confirmed by Ponceau S staining ((**right**) panel). One representative blot of at least three independent experiments is shown. (**b**) NAD^+^-quantitation assay. NAD^+^ was incubated with PTS1 in increasing concentrations with or without α-defensin-1 in the absence of Gαi. Decrease in fluorescence is a measure of NAD^+^-consuming enzymatic activity of PTS1, i.e., NAD^+^ is chemically converted in the end-point assay into a fluorescent molecule. Values are given as percent of control with NAD^+^ only, mean ± SD (n = 3 from one representative experiment out of three). Significance was tested using one-way ANOVA followed by Dunnett’s multiple comparison test and refers to controls without α-defensin-1 (** *p* ≤ 0.01, ns not significant).

Prompted by the in-silico findings of specific interaction interfaces for α-defensin-1 with PTS1, we investigated the in vitro interaction of α-defensin-1 with PTS1 and its substrate Gαi by dot blot analysis. In this assay, interaction partners were spotted onto a nitrocellulose membrane, which was then incubated with α-defensin-1 or PBS-T for a control. Then, bound α-defensin-1 was detected with a specific antibody (Figure 7a). The results revealed that α-defensin-1 binds to PTS1, but surprisingly even more pronounced to Gαi. To confirm that this interaction is independent of the N-terminal 6x His-tag present in Gαi and PTS1, His-tags were enzymatically removed. The same assay was conducted with the purified proteins and a comparable result was obtained (Figure 7a, middle row). Notably, α-defensin-1 is only bound to PTS1 but not the holotoxin PT in this assay. 

As we detected an interaction of α-defensin-1 with PTS1, we then analyzed whether α-defensin-1 can inhibit NADase activity of PTS1 in the absence of Gαi (Figure 7b). NAD^+^ was incubated with different concentrations of PTS1 with or without α-defensin-1 and the NAD^+^ consumption was measured by a fluorometric end-point assay, i.e., chemical conversion of NAD^+^ into a fluorescent molecule [40]. Except for causing higher NAD^+^ readings in the control wells without PTS1, α-defensin-1 had no significant impact on NADase activity of PTS1 (Figure 7b). This is in agreement with our results, obtained by computational modeling, that α-defensin-1 should not occlude the NAD^+^ binding site (Figure 6d).

In conclusion, α-defensin-1 and -5 both protect human cells from PT but use different molecular mechanisms to achieve this effect. While α-defensin-1 enters cells and neutralizes PT mainly by the inhibition of its enzyme activity, α-defensin-5 primarily inhibits the uptake of PT into cells.

## 3. Discussion

Pertussis is a severe disease with limited therapeutic options currently available [33,41]. Only during the early catarrhal stage of the disease, treatment with macrolide antibiotics is associated with an improvement of symptoms [42]. This presents a challenge as this stage occurs before the onset of the characteristic symptoms, such as whooping cough. Even though vaccination coverage is quite high, with ca. 81% of infants worldwide in 2021 [32], pertussis is still one of the leading causes of vaccine-preventable deaths and morbidity worldwide [43]. This precarious situation prompted us to investigate new therapeutic options against pertussis. In our approach, we directly target PT, which is a major contributor to pertussis pathology, including severe respiratory symptoms [21,29].

We demonstrated that α-defensin-1 and -5 both protect cells from intoxication with PT, though with different modes of inhibition. Uptake of PT into the cells is necessarily required for PT to reach its cellular target protein Gαi, and, therefore, to exert its mode of action. Inhibition by α-defensin-5 prevents this very first step of intoxication. In the presence of α-defensin-5, less PT was bound to the surface of cells, and less PT was taken up into cells. Contrarily, α-defensin-1 mainly inhibits the last step of PT intoxication, the enzymatic ADP-ribosylation of Gαi by PTS1. We showed that α-defensin-1 was taken up into cells, interacted with PTS1 in these cells and bound to PTS1 in-silico and in vitro. The predicted binding modes of α-defensin-1 and α-defensin-5 to PTS1 provide a rationale, at the molecular level, for the different experimental behaviors of the α-defensins 1 and 5. The lack of a preferred binding interface in the α-defensin-5/PTS1 system, in contrast to the much favored and defined interactions of α-defensin-1 with PTS1, is in agreement with the experimental in vitro observation of α-defensin-1 as a stronger inhibitor of PTS1 with respect to α-defensin-5. These different modes of action might complement each other in a therapeutic context.

Human defensins contain six cysteine residues, which form three intramolecular disulfide bonds. Up to now, there are six different human α-defensins, which are classified based on their site of production: Human neutrophil peptides 1 to 4 (HNP 1–4 = α-defensin-1-4), found in primary granules of neutrophils, and human enteric defensins 5 and 6 (HD 5–6 = α-defensin-5-6) found in Paneth cells in the crypts of the small intestine. Notably, α-defensin-4 is found in neutrophils in substantially lower concentrations compared with α-defensin-1-3 [36], and α-defensin-1-3 only differ in their first out of 30 amino acid residues. In light of these observations and considering the lack of cellular protection from PT by α-defensin-6, we focused our investigations on α-defensin-1 and -5.

Defensins are mostly known for their antimicrobial activity. Like most antimicrobial peptides, the defensins interact and disrupt the lipid membranes of microbes, leading to their lysis [44]. In addition to their antimicrobial activity, human α-defensins inhibit several bacterial toxins with different modes of action. As an example, α-defensin-1 exhibited inhibitory effects on enzyme activity of *B. anthracis* lethal factor (LF) [45], *Diphtheria* toxin (DT) and *Pseudomonas* exotoxin A (ETA) [46]. α-Defensin-5 has been shown to neutralize *C. difficile* toxin A (TcdA) by the formation of inactive toxin–defensin aggregates and *C. difficile* transferase toxin (CDT) by influencing the binding and transport component [47]. However, α-defensin-5 had no influence on enzyme activity of TcdA or CDT. The pore formation of CDT by its B-component was impaired by α-defensin-1 and -5 [47,48], similar to the mechanism shown for *Clostridium perfringens* iota toxin [49]. Another reported mechanism of inhibition is the local unfolding of *V. cholerae* and *Aeromonas hydrophilia* MARTX effector domains, which leads to their thermal unfolding, increased susceptibility to proteolysis and precipitation, observed for α-defensin-1 and also to a lesser degree for α-defensin-5 [50]. PTS1 is also known to be thermally unstable, which is necessary for its transport from the ER to the cytosol [12], making this another conceivable inhibitory mechanism. A common mechanism of inhibition of bacterial toxins by α-defensins could not be determined so far.

Interestingly, α-defensin-1 was also shown to be a substrate for the human arginine-specific ADP-ribosyltransferase 1 (ART1) [51,52]. Consequently, we investigated whether α-defensin-1 might also be a substrate of PTS1. For this, α-defensin-1 was incubated with PTS1 for 40 min at room temperature and then analyzed by mass spectrometry. No ADP-ribosylation or any other modification of α-defensin-1 or α-defensin-5 by PTS1 was detected.

The small size of the defensins and their diverse beneficial effects make them promising candidates for drug development. Therapeutic peptides have gained significant attention over the last years, with nearly 20 new peptide-based clinical trials annually and over 60 peptide drugs already approved [53]. An advantage of their small size compared to antibodies or other proteins is their ability to bind deep into folding pockets of proteins and to reach cellular targets [53]. Furthermore, endogenous peptides, such as defensins are usually less immunogenic and therefore better tolerated by patients than foreign agents [41,53,54]. Our finding that α-defensin-1 is taken-up into all tested cell lines serves our aim of using it as a therapeutic drug reaching intracellular targets. This finding is in line with previous studies of other groups, which found that α-defensin-1 accumulates intracellularly in human cervical CaSki cells and inhibits human simplex virus, also post-entry [55] and is also taken up into bone marrow-derived macrophages [56]. While α-defensin-5, found in the intestine, does not physiologically come into contact with PT, which mainly acts in the respiratory tract, it might have therapeutic implications as an inhibitor of PT uptake into cells.

The identification of α-defensin-1 and -5 as inhibitors of PT and the characterization of their underlying mechanism of inhibition contributes to a better understanding of innate immune responses to PT. Our computational studies deliver a rationale for the mechanisms of inhibition at the molecular level as well as an explanation for the different behaviors of α-defensin-1 with respect to α-defensin-5, based on their interactions with PTS1. Taken together, our findings will allow for the optimization of α-defensins as inhibitors of PT, paving the way for the development of novel therapeutic strategies against whooping cough.

## 4. Materials and Methods

### 4.1. Compounds and Reagents

*Bordetella pertussis* toxin (PT) was purchased from Sigma-Aldrich, Merck (Darmstadt, Germany). Recombinant Gαi and recombinant PTS1 were expressed and purified as described earlier (Ashok et al. [40]). The peptides α-defensin-1, -2, -3, -4, -5 and -6 and β-defensin-1 and -2 were purchased from PeptaNova (Sandhausen, Germany). Linearized α-defensin-1 and -5 (cysteine residues changed to serine residues in their amino acid sequence) were custom-made and purchased from PSL (Heidelberg, Germany). For the NADase activity assay, α-defensin-1 was purchased from ProteoGenix (Schiltigheim, France) and further refolded and purified (see Supplementary Material Figures S5 and S6).

### 4.2. Cell Lines

Materials for cell culture were all purchased from Gibco (Thermo Fisher Scientific, Waltham, MA, USA) unless indicated otherwise. A549 cells (human lung adenocarcinoma cells; ATCC^®^ Manassas, VA, USA), CaCo-2 cells (human epithelial colorectal adenocarcinoma cells, HTB-37; ATCC, Manassas, VA, USA) and J774A.1 cells (murine macrophage-like cells; DSMZ, Braunschweig, Germany) were cultivated in DMEM, Vero cells (African green monkey kidney cells; DSMZ, Braunschweig, Germany) and HeLa cells (cervical carcinoma cells; DSMZ, Braunschweig, Germany) were cultivated in MEM. All media were supplemented with 10% FCS, 1 mM sodium pyruvate, 0.1 mM non-essential amino acids and 100 U/mL penicillin and 100 g/mL streptomycin. CHO-K1 cells (Chinese hamster ovary cells strain K1; DSMZ, Braunschweig, Germany) were cultivated in DMEM and HAM’s F12 (1:1), supplemented with 5% FCS, 1 mM sodium pyruvate, 0.05 mM non-essential amino acids and 100 U/mL penicillin and 100 g/mL streptomycin. Cells were cultivated under humidified conditions at 37 °C with 5% CO_2_ and trypsinized and reseeded every two to three days for at most 25 times. For intoxication experiments, cells were seeded in culture dishes one day before and treated in FCS-free media with PT and the respective compounds.

### 4.3. Sequential ADP-Ribosylation of Gαi in Lysates from Toxin-Treated Cells

Cells were seeded into 24-well plates and treated with PT and defensin or H_2_O (solvent of defensins) as a control in FCS-free medium. After 4 h, cells were washed to remove unbound protein and frozen for cell lysis. ADP-ribosylation buffer (0.1 mM Tris-HCl (pH 7.6), 20 mM DTT, 0.1 μM ATP and protease inhibitor complete (Roche)) was added and cell lysates were transferred to reaction tubes. For sequential in vitro ADP-ribosylation of Gαi, which had not been modified by PT in the cells during intoxication, recombinant PTS1 (50 nM) and biotin-labeled NAD^+^ (1 μM; R&D Systems, Abingdon, UK) were added and incubated for 40 min at room temperature. Then, samples were subjected to SDS-PAGE and Western blotting with streptavidin–peroxidase (Strep–POD, Sigma-Aldrich, Merck, Darmstadt, Germany) for detection of biotin-labeled and thus sequentially ADP-ribosylated Gαi. Quantification of signals was performed using ImageJ software v.1.52a (National Institutes of Health, Bethesda (NIH)). As a loading control, Hsp90 (primary antibody from Santa Cruz Biotechnology, Dallas, TX, USA) signals were detected.

### 4.4. In Vitro Enzyme Activity of PTS1

Recombinant PTS1 (50 nM = 1.17 μg/mL) was incubated with recombinant Gαi (0.5 μM = 20.2 μg/mL) and biotin-labeled NAD^+^ (1 μM; R&D Systems) in a 50 nM sodium phosphate buffer (pH 7.0) in the presence of the respective defensin or H_2_O as a control for 40 min at room temperature. After that, samples were subjected to SDS-PAGE and Western blot analysis with streptavidin-peroxidase (Strep-POD, Sigma-Aldrich, Darmstadt, Germany) for detection of biotin-labeled and thus enzymatically modified Gαi. Quantification of signals was performed using ImageJ software v.1.52a (NIH). To control equal loading of samples, Ponceau-S-staining was used.

### 4.5. Flow Cytometry Binding Assay

Cells were detached from culture dishes using 25 mM EDTA in PBS and resuspended in FCS-free medium. The cells (1 × 10^5^ in 0.2 mL per sample) were incubated with defensin and 488-labeled PT for 15 min on ice to allow binding of PT to cells but no internalization. Cells were washed twice by centrifugation to eliminate unbound 488-labeled PT. Fluorescence of cells was measured using a BD FACS Celesta™ flow cytometer (Becton, Dickinson and Company, Franklin Lakes, NJ, USA) and the BD FACSDiva^TM^ software 8.0.1.1. DyLight488 excitation was performed with a blue laser (488 nm) and emitted fluorescence detected with a 530 nm (530/30) bandpass filter. Data analysis of gated cell populations was performed using Flowing Software v2.5.1. (Turku Centre of Biotechnology, Finland). PT was labeled with DyLight^®^ 488 NHS Ester (Thermo Fisher Scientific, Rockford, IL, USA) according to the manufacturer’s protocol and excess dye was removed with Zeba^TM^ Spin Desalting Columns (7K MWCO, Thermo Fisher Scientific, Waltham, MA, USA).

### 4.6. Immunostaining and Fluorescence Microscopy

Cells were seeded in 8- or 18-well μ-slides (ibidi GmbH, Gräfelfing, Germany) and grown for one day. After respective treatments in FCS-free medium, cells were washed with PBS, fixed with 4% paraformaldehyde for 20 min, permeabilized with 0.4% (*v*/*v*) Triton X-100 in PBS for 5 min, quenched by glycine (100 mM in PBS) for 2 min and blocked with 10% NGS (Jackson ImmunoResearch, West Grove, PA, USA) and 1% BSA in PBS containing 0.1% Tween 20 (PBS-T) for 1 h at 37 °C. Subsequently, cells were incubated with primary antibodies (anti-PTS1, Santa Cruz; anti-PT, Abcam; anti-HNP and anti-α-defensin-5, Santa Cruz) and fluorescence labeled secondary antibodies in blocking solution each for 1 h at 37 °C. Nuclei were stained with Hoechst 33342 (1:10,000, Thermo Fisher Scientific, Waltham, MA, USA) for 5 min. Cells were washed with PBS-T in between individual steps. Images were obtained using an iMIC digital microscope (FEI Munich, Munich, Germany) and Live Acquisition 2.6 software (FEI Munich) and processed using ImageJ software v.1.52a (NIH).

### 4.7. Cell Viability Assay

CHO-K1 cells were seeded in 96-well plates and incubated with defensin or H_2_O as a control for 24 h. DMSO (20%) was used as a positive control for causing cell death. Cell Titer 96^®^ Aqueous One Solution (MTS assay, Promega, Walldorf, Germany) was then added to cells and incubated for 1 h at 37 °C to determine cell viability. Absorbance was measured at 490 nm via a microtiter plate reader.

### 4.8. Protein Interaction Analysis in Cells Using Proximity Ligation Assay

Cells were seeded in 18-well μ-slides (ibidi GmbH, Gräfelfing, Germany) and incubated with PT and defensin for 4 h at 37 °C. Afterwards, cells were washed with PBS, fixed with 4% PFA, permeabilized and blocked with 10% NGS and 1% BSA in PBS-T. Then, cells were incubated with rabbit anti-PT (Abcam) and mouse anti-HNP (= α-defensin-1) (Santa Cruz) or mouse anti-α-defensin-5 (Abcam) primary antibodies for 1 h at 37 °C. PLA was performed according to the manufacturer’s protocol (Duolink using PLA technology, Sigma-Aldrich, Merck). In brief, cells were incubated with PLA secondary antibodies (anti-rabbit for detecting anti-PT and anti-mouse for detecting anti-defensin) having attached specific oligonucleotide sequences to each. If they get in close proximity, they can form a ring structure. By addition of ligase and polymerase, rolling circle amplification can occur. Samples were probed with fluorescence labeled oligonucleotides, complementary to the amplification product for detection of the protein interaction. PLA signals were counted from fluorescence images using ImageJ software v.1.52a (NIH).

### 4.9. In Silico Modeling of PTS1/Defensin Complexes

For the sequence-based predictions of interaction likelihood, the sequences of the recombinant PTS1, α-defensin-1, and α-defensin-5, were split into overlapping subsequences of 20 residues each. We used the PPI-Detect tool [37] as implemented in its web server (https://protdcal-suite.cbe.bci.tu-dortmund.de/ppi_detect/) (accessed on 12 May 2023) [57] for the predictions, considering the top-10 interacting sequence pairs inside the applicability domain (AD). The interacting sequence pairs were mapped onto the structure of PTS1 (PDB ID 7SNE [39]), α-defensin-1 (PDB ID 3GNY [58]), α-defensin-5 (PDB ID 1ZMP [59]).

PTS1/α-defensin-1 and PTS1/α-defensin-5 complexes were modeled with AlphaFold Multimer [38] using the sequences of recombinant PTS1, α-defensin-1 and α-defensin-5 as inputs. The best-scored model was then used for unbiased MD simulations where the center of masses of PTS1 and the defensins were 40 Å apart. Three different initial configurations were set for each defensin. The systems were solvated, using the TIP3P water model [60], in a cubic box with a minimum distance of 14 Å between the protein and the edge of the box. Periodic boundary conditions were applied. Na^+^ and Cl^−^ ions were added to the box for neutralization and, in addition, to reach a concentration of 150 mM. The protein topology was set using the CHARMM36 force field [61]. The systems were minimized using a steepest descent algorithm [62] until the maximum force reached 100 kJ/mol.nm. Then, a two-step minimization, comprising a 200 ps equilibration step in the NVT ensemble, followed by 2 ns of equilibration in the NPT regime, was carried out. Protein heavy atoms were fixed during the equilibration using a 1000 kJ/mol force restraint. Finally, three replicas of 1.5 μs production MD simulations were performed without any restraints. The integration of Newton’s equations of motion was performed using a leap-frog algorithm with a 2 fs time step [63]. The temperature and the pressure were maintained at 298 K and 1 bar by using the V-rescale temperature thermostat [64] and C-rescale barostat [65]. The bonds of the peptide and the osmolyte molecules were constrained by the LINCS algorithm [66]. The nonbonded Lennard–Jones interactions were cut-off at 12 Å. Electrostatic interactions were calculated using the particle mesh Ewald (PME) method [67] with a cut-off of 12 Å and a grid-spacing of 1.6 Å. All the runs were performed using the Gromacs 2022 package [68].

Trajectory frames were grouped into clusters using the Daura’s et al., algorithm [69] with a RMSD cutoff of 5 Å. For each cluster, the structure with the smallest average RMSD from all other members of the cluster was selected as representative of the cluster. Contacts between PTS1 and the defensins were defined using a minimum distance cutoff of 4 Å between the atoms of both proteins. The contact frequency per residue was calculated as the number of frames where the residue is establishing contacts with the other protein divided by the total number of frames analyzed. The structures were visualized with Pymol 2.6 (PyMOL Molecular Graphics System, Version 2.6 Schrödinger, LLC).

### 4.10. Protein Interaction Analysis In Vitro Using Dot Blot System

Decreasing amounts of purified proteins were vacuum-aspirated onto a nitrocellulose membrane using the dot blot system (Bio-Rad, Feldkirchen, Germany) according to the manufacturer’s instructions. Transfer of proteins was confirmed by Ponceau-S-staining. Then, the membrane was blocked with 5% skim milk powder in PBS-T. Membrane was cut and probed with defensin or PBS-T as a negative control for 1 h. After extensive washing (5 × 5 min), membrane was incubated with anti-HNP antibody and peroxidase-coupled anti-mouse antibody for detection of bound α-defensin-1 by using the ECL system. His-Tag was detected using anti-6x His antibody.

### 4.11. NADase Activity Assay

The 50 μL reactions were conducted in a flat bottom 96-well black plate (Greiner Bio-One, Polypropylene 96-well F-bottom Microplates, Fisher Scientific, Schwerte Germany) in 100 mM HEPES, 500 mM sodium chloride, 10% (*w*/*v*) glycerol, 0.5 mM TCEP (pH 7.5). The concentration of PTS1 was titrated at 0.5, 1, 5, and 10 μM while keeping the NAD^+^ and α-defensin-1 concentrations constant at 10 μM and 12 μM, respectively. PTS1 was incubated with α-defensin-1 for 15 min before addition of NAD^+^ as the final reagent. The reactions were incubated for 1 h at room temperature with shaking at 300 rpm. To stop the reaction, and to convert the remaining NAD^+^ to a fluorescent molecule, 20 μL of 2 M KOH and 20 μL of 20% acetophenone (Sigma, diluted with ethanol) were added, followed by incubation at room temperature for 10 min. Subsequently, 90 μL of formic acid (Fisher Scientific) was added, and the reaction mixture was further incubated for 20 min at room temperature. The fluorescence readings were recorded with the VICTOR Nivo multimode plate reader (PerkinElmer, Waltham, MA, USA), with excitation and emission wavelengths set at 355 nm (±20 nm) and 450 nm (±5 nm), respectively.

## Data Availability

The datasets generated during and/or analyzed during the current study are available from the corresponding author on reasonable request.

## Supplementary Materials

The following supporting information can be downloaded at: https://www.mdpi.com/article/10.3390/ijms241310557/s1. Reference [70] is cited in the supplementary materials.
