# Supplementary material for: Inhibition of Pertussis Toxin by Human α-Defensins-1 and -5: Differential Mechanisms of Action
Source: Int J Mol Sci. 2023 Jun 23;24(13):10557. doi: 10.3390/ijms241310557 (PMC10341622; doi:10.3390/ijms241310557)
Supplement: Supplementary file 1 [file ijms-24-10557-s001.zip › 2023-06-08_Supplementary material.pdf]

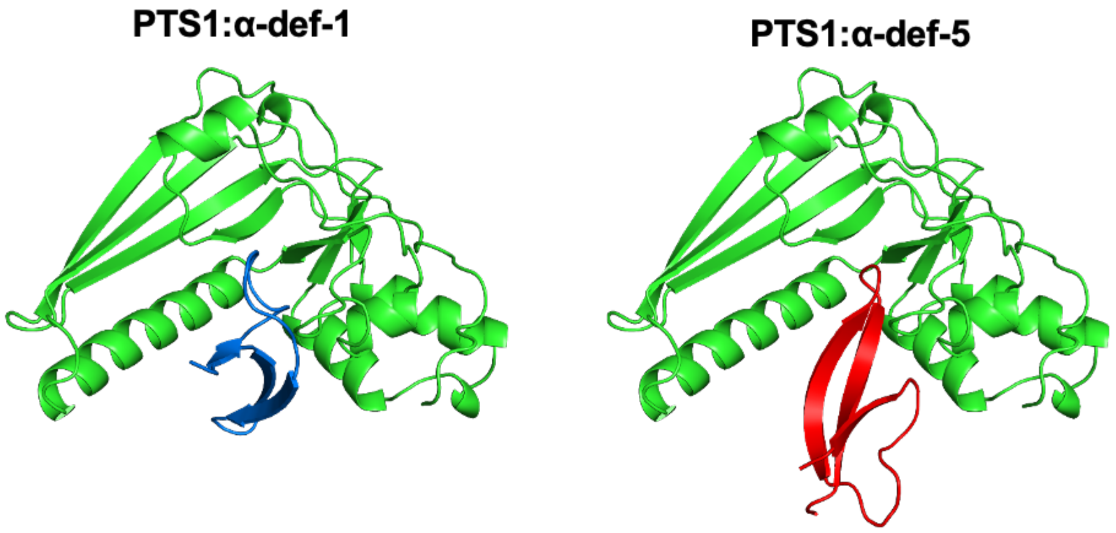

Supplementary Figure S1. 3D representation of the top model of the PTS1/α-defensin-1 (left) and PTS1/α-defensin-5 (right) complexes generated with AlphaFold Multimer. PTS1 is shown in green, α-defensin-1 in blue, and α-defensin-5 in red.

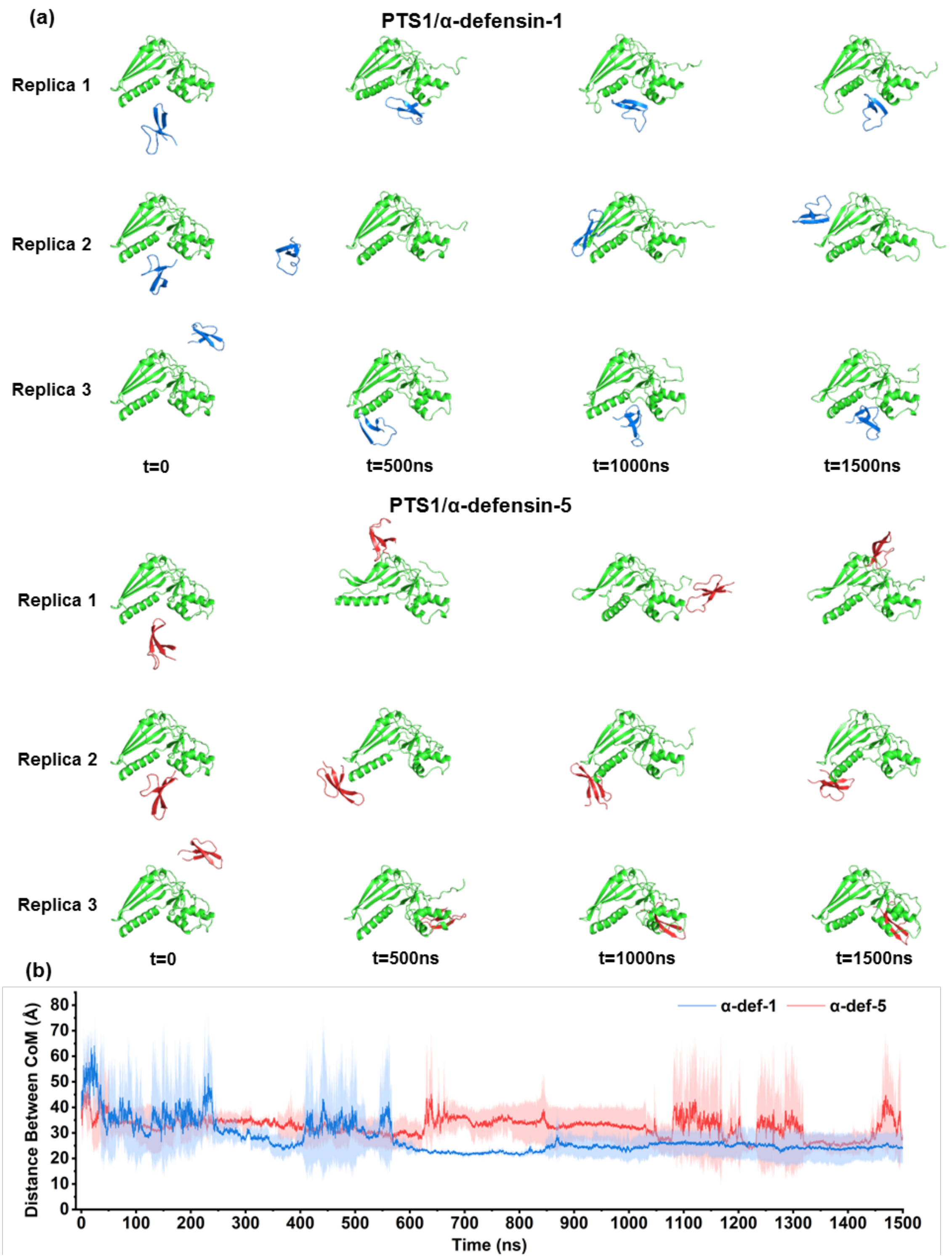

Supplementary Figure S2. (a). Selected frames at 0, 500, 1000 and 1500 ns of the three replicas (each 1.5 μs) of PTS1/α-defensin-1 and PTS1/α-defensin-5 MD simulations. PTS1 is represented in green, α-defensin-1 in blue, and α-defensin-5 in red. (b) Average of distance between the center of mass (CoM) of PTS1 and α-defensin-1 (blue) or α-defensin-5 (red) over the three replicas of the PTS1/α-defensin-1 and PTS1/α-

defensin-5 MD simulations. The lighter color shade represents the standard deviation of the distance between the CoMs for the three replicas.

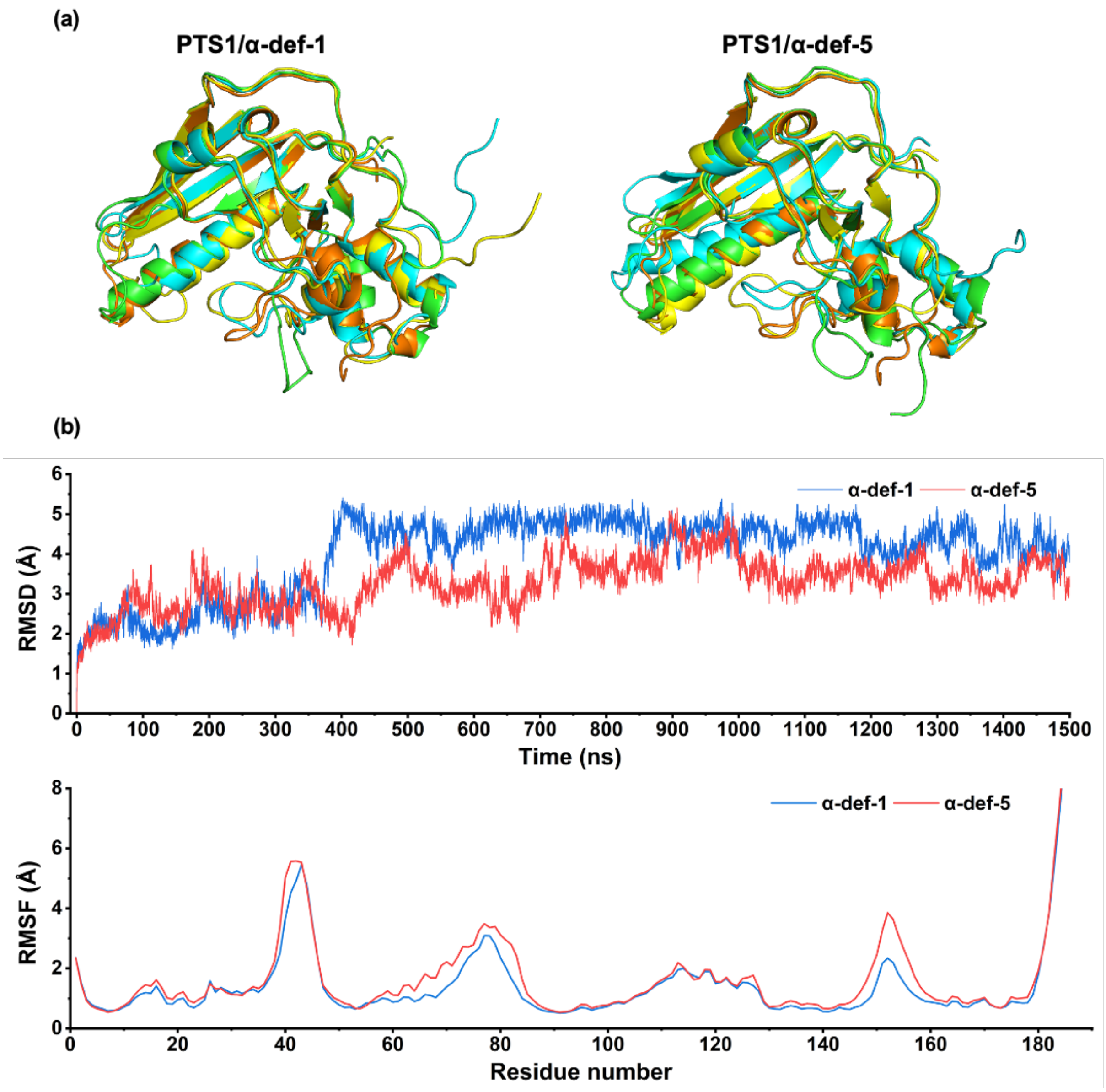

Supplementary Figure S3. a) Superposition of the 3D structure of PTS1 modelled by AlphaFold (orange) and the central structure of the 3 most populated clusters (green, cyan and yellow for cluster 1, 2 and 3, respectively) for the combined last 800 ns of the three replicas of the PTS1/α-defensin-1 system (left) and PTS1/α-defensin-5 (right).The RMSD of the α-carbons of PTS1 after superposition onto the AlphaFold model is 0.947 Å for cluster 1, 0.913 Å for cluster 2 and 1.015 Å for cluster 3 of the PTS1/α-defensin-1 simulations. For PTS1 in the PTS1/α-defensin-5 simulations, the RSMDs are: 0.968 Å for cluster 1, 0.858 Å for cluster 2 and 1.155 Å for cluster 3. **(b)** Top: Average root mean square deviation (RMSD) of the backbone of PTS1 over the three replicas of the

PTS1/α-defensin-1 (blue) and PTS1/α-defensin-5 (red) MD simulations. Bottom: Average root mean square fluctuation (RMSF) of the backbone of PTS1 in the combined last 800 ns of the three replicas of the PTS1/α-defensin-1 (blue) and PTS1/α-defensin-5 (red) MD simulations.

Refolding, purification, and analysis of synthetic alpha defensin 1.

Refolding of alpha-defensin 1

The refolding was performed according to a previous work [1]: synthetic alpha-defensin-1 was dissolved at 0.5 mM in 187.3 mL 0.1% TFA and then diluted with a mixture of 193.3 mL 2-Propanol + 193.3 mL acetonitrile. A 490 µL-aliquot of 25% ammonia was added, and the mixture was stirred for 16 hours at room temperature. The solvent was removed in a SpeedVac vacuum concentrator.

Semipreparative RP-HPLC

The dry material was dissolved in 10 mL of 10% acetic acid and fractionated by reversed-phase HPLC on a Luna C18 column (Phenomenex, USA) of dimensions 21.2 x 250 mm and particle size of 5 µm. The separation was performed at a flow rate of 12.23 mL/min using the gradient program (min/%B) 0/5, 5.09/5, 28.71/25, 49.55/50, and 70.39/100, being A, 0.1%TFA in water, and B, 0.1% TFA in acetonitrile. Elution was monitored online at 280 nm (see supplementary figure I). Fractions were collected every minute and dried in a SpeedVac vacuum concentrator.

Mass spectrometry analysis

Samples from the semipreparative purification were analyzed by an Axima Confidence MALDI-TOF MS (Shimadzu, Japan) in positive linear mode on a 384-spot stainless-steel sample plate. Spots were coated with 1 µL 5 mg/mL CHCA previously dissolved in matrix diluent (Shimadzu, Japan), and the solvent was allowed to air dry. Then 0.5 µL sample or standard was applied onto the dry pre-coated well and immediately mixed with 0.5 µL matrix; the solvent was allowed to air dry. All spectra were acquired in the positive ion linear mode using a 337-nm N_2 laser. Ions were accelerated from the source at 20 kV. A hundred profiles were acquired per sample, and 20 shots were accumulated per profile. The equipment was calibrated with a standard mixture provided in the TOFMix™ MALDI kit (Shimadzu, Japan). Measurements and MS data processing were controlled by the MALDI-MS Application Shimadzu Biotech Launchpad 2.9.8.1 (Shimadzu, Japan). GraphPad Prism 9 (GraphPad Prism Software LLC, San Diego, CA, USA) was used for figure preparation. See supplementary figure II.

Analytical RP-HPLC

Fractions yielding the expected average mass corresponding to the refolded peptide with disulfide bridges were dissolved in 10 mL of 10% acetic acid and analyzed by

reversed-phase HPLC on an XSelect™ PREMIER Peptide CSH C18 HPLC column (Waters, USA) of dimensions 4.6 x 50 mm and particle size of 2.5 μm. The separation was performed at a flow rate of 0.8 mL/min using the gradient program (min/%B) 0/5, 1/5, 2/10, 3/10, 15/45, 20/100, being A, 0.1%TFA in water, and B, 0.1% TFA in acetonitrile. Elution was monitored online at 214 nm. See supplementary figure III.

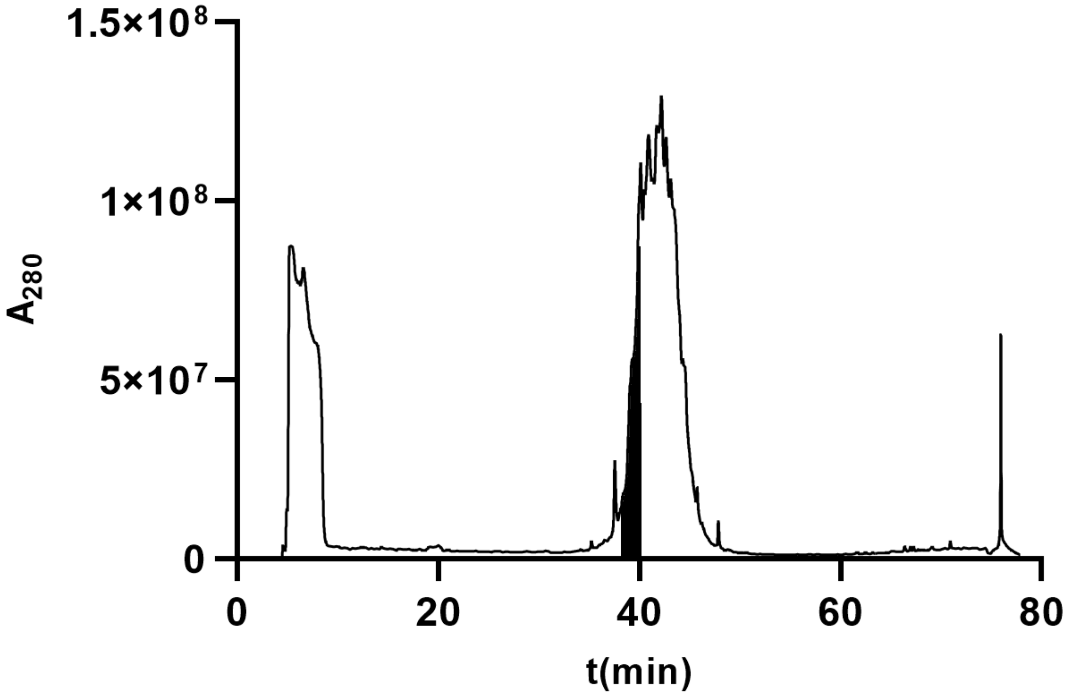

Supplementary Figure S4. Purification of the refolded synthetic alpha defensin 1 by reversed-phase C18 HPLC. Refolded high-purity alpha defensin 1 was found in the highlighted region of the chromatographic profile.

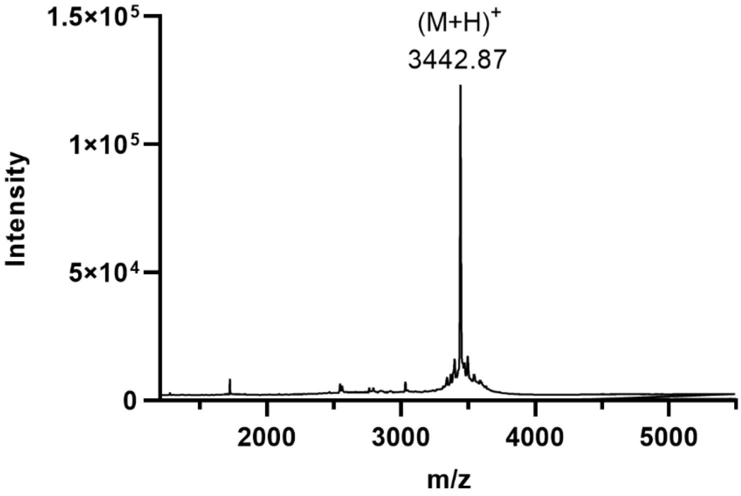

Supplementary Figure S5. a) MALDI-TOF spectrum of the refolded synthetic alpha defensin 1. The experimental average mass of 3441.86 Da closely matches the theoretical one of 3442.093 Da for the peptide with three disulfide bridges.

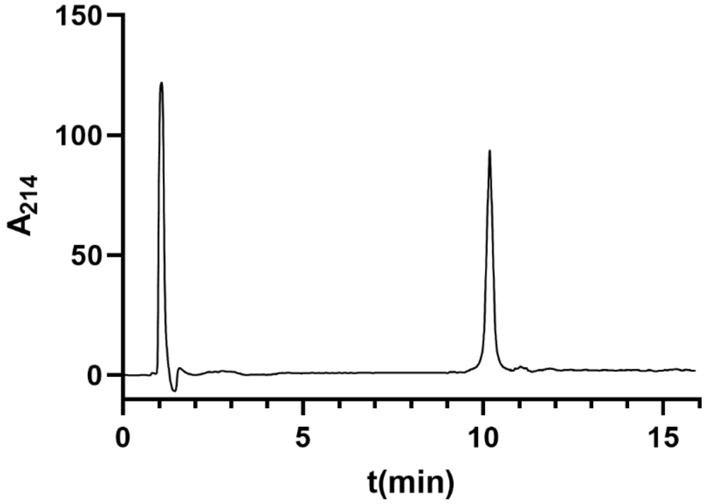

Supplementary Figure S6. RPC18-HPLC analysis of the high purity fraction containing refolded synthetic alpha defensin 1.

References

1. Vicente, F.E.M.; González-Garcia, M.; Diaz Pico, E.; Moreno-Castillo, E.; Garay, H.E.; Rosi, P.E.; Jimenez, A.M.; Campos-Delgado, J.A.; Rivera, D.G.; Chinea, G.; et al. Design of a Helical-Stabilized, Cyclic, and Nontoxic Analogue of the Peptide Cm-P5 with Improved Antifungal Activity. *ACS Omega* **2019**, *4*, 19081–19095, doi:10.1021/acsomega.9b02201.
